# Peripheral Sensory Nerve Tissue but Not Connective Tissue Is Involved in the Action of Acupuncture

**DOI:** 10.3389/fnins.2019.00110

**Published:** 2019-02-20

**Authors:** Suchan Chang, O. Sang Kwon, Se Kyun Bang, Do-Hee Kim, Min Won Baek, Yeonhee Ryu, Jong Han Bae, Yu Fan, Soo Min Lee, Hyung Kyu Kim, Bong Hyo Lee, Chae Ha Yang, Hee Young Kim

**Affiliations:** ^1^College of Korean Medicine, Daegu Haany University, Daegu, South Korea; ^2^Clinical Medicine Division, Korea Institute of Oriental Medicine, Daejeon, South Korea; ^3^Department of Physics, Yeungnam University, Gyeongsan, South Korea

**Keywords:** acupuncture, peripheral sensory nerve, connective tissue, robotic acupuncture needle twister, collagenase

## Abstract

Acupuncture has been used to treat a variety of diseases and symptoms for more than 2,500 years. While a number of studies have shown that nerves are responsible for initiating the effects of acupuncture, several lines of study have emphasized the role of connective tissue in the initiation of acupuncture signals. To determine whether nerves or connective tissue mediate the action of acupuncture, we constructed a robotic acupuncture needle twister that mimicked the twisting of the needle by an acupuncturist, and we examined the role of nerves and connective tissues in the generation of acupuncture effects in rat cocaine-induced locomotion, stress-induced hypertension, and mustard oil-induced visceral pain models. Robotic or manual twisting of acupuncture needles effectively suppressed cocaine-induced hyperactivity, elevated systemic blood pressure or mustard oil-induced visceral pain in rats. These acupuncture effects were completely abolished by injecting bupivacaine, a local anesthetic, into acupoints. However, disruption of connective tissue by injecting type I collagenase into acupoints did not affect these acupuncture effects. Our findings suggest that nerve tissue, but not connective tissue, is responsible for generating the effects of acupuncture.

## Introduction

Acupuncture has been used to treat various diseases in Asian countries over the last 2,500 years and has been widely practiced in many countries. In 1997, the United States' National Institutes of Health (NIH) reported acupuncture's safety and efficacy for treating a wide range of conditions, including drug abuse, stroke rehabilitation, and headache (Nih Consensus Conference, 1998). In a bibliometric review of the publications on acupuncture research in PubMed over the last 20 years, acupuncture research has increased markedly in the past two decades, with twice the growth rate of overall biomedical research. While pain (approximately 38% of publications) is consistently the most common topic of acupuncture research, the publications of randomized clinical trials have been increasing in numbers at a high rate (Ma et al., 2016). Although these studies have shown acupuncture's effectiveness, the mechanisms by which the acupuncture signals are initiated and conveyed remain contradictory and inconclusive.

Acupuncture is a therapeutic intervention for improving the efficiency of homeostatic mechanisms of the body by stimulating acupoints. There are two leading models of the initiation of acupuncture signals: the neurologic model and the connective tissue model. The neurologic model, which is by far the more widely accepted and studied, posits mediation by the nervous system, with acupuncture signals being initiated by the activation of sensory nerve endings and specific nerve fibers and transmitted to the brain through nervous pathways, resulting in a variety of physiological effects. In support of this view, it was reported that acupuncture produces an antinociceptive effect by releasing adenosine at peripheral nerve terminals in rodents (Goldman et al., 2010). Acupuncture stimulation at acupoints in the wrist, PC5-6, increased Aδ- and C-fiber activity to evoke cardiovascular effects (Zhou et al., 2005). Our previous studies showed that acupuncture at HT7 attenuates drug-induced or drug-seeking behaviors for substances such as cocaine, morphine and ethanol (Yoon et al., 2004, 2010, 2012; Yang et al., 2010). During acupuncture stimulation, peripheral sensory afferents, such as Pacinian and Meissner's corpuscles, are activated, and the afferent signals are transmitted via the A-fibers of the ulnar nerve (Kim et al., 2013) and the somatosensory pathway in the spinal dorsal column (Chang et al., 2017). While cumulative evidence has supported the neurological model, in the last two decades another hypothesis, known as the “connective tissue model,” has emerged to address possible roles of connective tissue in the initiation of acupuncture's signals (Langevin and Yandow, 2002; Langevin, 2014). According to this model, twisting or rotation of the acupuncture needle creates winding of connective tissue around the needle and deformation of collagen fibers, which initiates mechanical signals transmitted via the whole-body fascia network, the layers of connective tissue surrounding muscle, organs, and blood vessels. Research groups have proposed that the connective tissue may mediate the effects of manual acupuncture through a number of pathways: transmission of a mechanical signal from the needle to nearby sensory afferent nerves, potential purinergic signaling within connective tissue fibroblasts with local effects on sensory nerves, and direct effects on connective tissue pathology, possibly involving reductions in inflammation and/or fibrosis (Langevin and Yandow, 2002; Langevin et al., 2007; Langevin, 2014; Wu et al., 2015). However, it remains to be established whether connective tissue mediates the therapeutic effects of acupuncture. While these models have been thought to provide plausible explanations for the initiation of acupuncture signals, evidence is currently lacking as to whether these two models work in separate or synergistic ways.

To explore whether acupuncture effects are mediated through nerve or connective tissue, we used our newly constructed robotic needle twister to test the effects of twisting acupuncture in the rat models of cocaine-induced locomotion, immobilization-induced hypertension and mustard oil-induced visceral pain, and explored whether the effects of acupuncture are altered by a blockade of nerves or disruption of connective tissue.

## Materials and Methods

### Animals

Male Sprague-Dawley rats (weight 270–320 g, Daehan Animal, Korea) were used, and each group consisted of 5–8 rats. Animals were housed at a constant humidity (40~60%) and temperature (22 ± 2°C), with a 12 h/12 h light/dark cycle and allowed free access to food and water until use. All procedures were carried out in accordance with the NIH Guide for Care and Use of Laboratory Animals and approved by the Institutional Animal Care and Use Committee (IACUC) at Daegu Haany University.

### Chemicals

Cocaine (15 mg/ml saline, MacFarlan Smith Ltd., UK), mustard oil (30~50 μl; allyl isothiocyanate, Sigma Aldrich, USA), and bupivacaine (20 μl/loci, 5 mg/ml, Huons Pharm, South Korea; a long-acting local anesthetic) were used. Type I collagenase (20 μl/loci, 5 mg/ml saline, Sigma Aldrich, USA) or together with toluidine blue (20 μl/loci, 1 mg/ml, Sigma Aldrich) was injected. Bupivacaine or type I collagenase was injected into acupuncture points to a depth of 3 mm, perpendicular to the skin surface, by using a syringe with a 32-gauge needle.

### Measurement of Rotational Duration and Angle During Manual Needle Twisting

**Figure 1:**
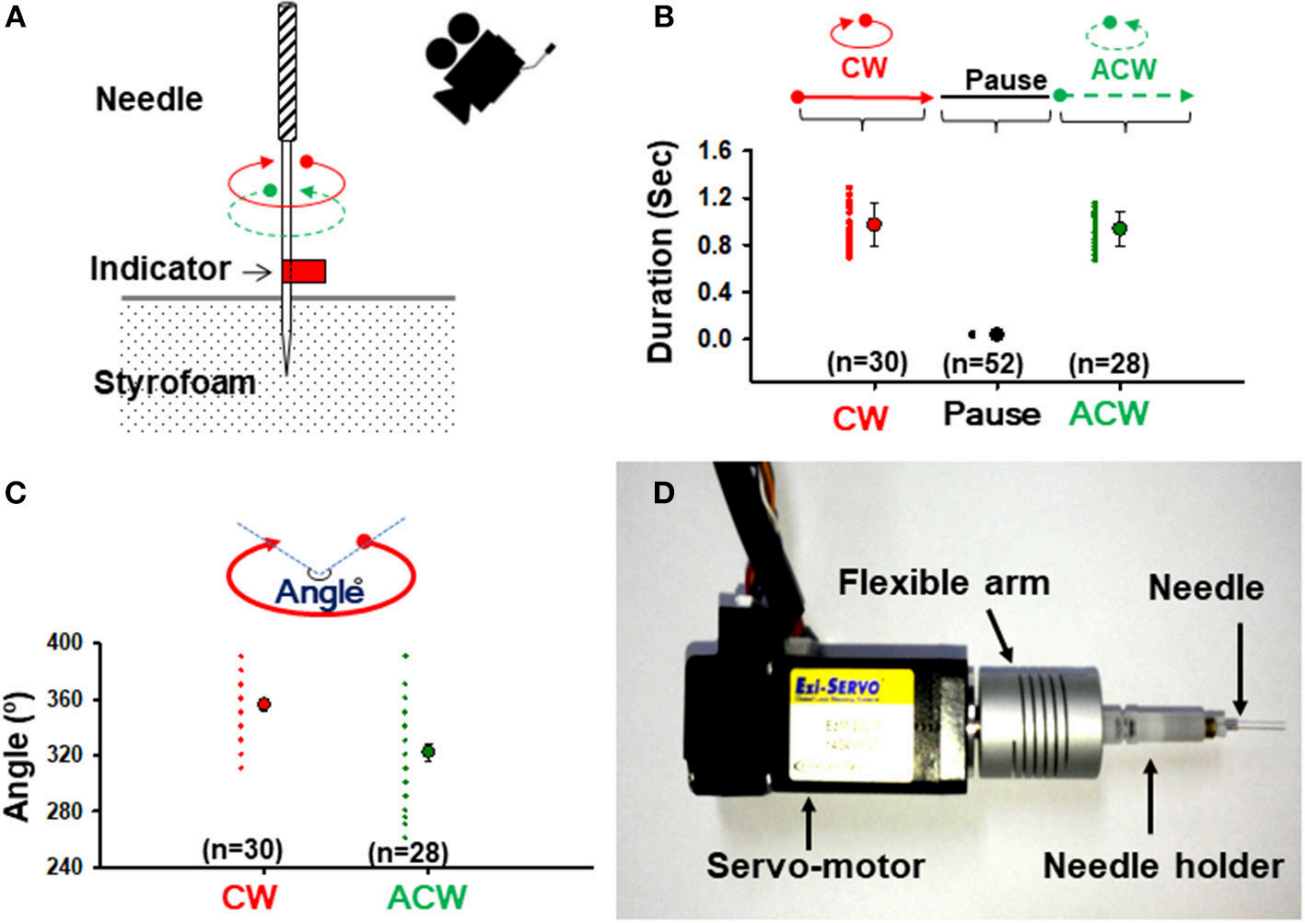
Twisting acupuncture and construction of a robotic acupuncture needle twister (RANT). **(A)** Measurement of rotational durations and angles during manual needle twisting. A needle with a red tape on the shaft was inserted into a Styrofoam board and video-recorded. **(B)** Mean duration of needle rotations. The motions of 30 CW turns, 52 pauses and 28 ACW turns during needle twisting by 3 acupuncturists were analyzed. **(C)** Mean rotation angle of the needle. The motions of 30 CW turns and 28 ACW turns during needle twisting by 3 acupuncturists were analyzed. **(D)** A constructed RANT device. The servo motor in the needling instrument was fitted to a flexible arm fixed with a needle holder and controlled by the control software. CW, clockwise direction; ACW, anticlockwise direction.

A needle (0.20 mm in diameter, needle length of 30 mm and handle length of 20 mm; Dongbang Medical Co., Korea) with red tape on the shaft was inserted into a Styrofoam board to measure the rotational duration (speed) and angle during manual needle twisting (Figure 1A). Acupuncturists (*n* = 3) were asked to twist the handle of the acupuncture needle for at least 20 s and video recorded. This procedure was repeated two times per person. Rotation duration and angle were analyzed using a media player program (Gom Player, Gom and Company, Korea).

### A Robotic Acupuncture Needle Twister (RANT) and Acupuncture Treatment

A robotic twister was constructed to mimic a needle twisting technique that is commonly performed by acupuncturists. This device consisted of a handheld needling instrument coupled to a servo motor (ez-SERVO, Fastech, Korea), a personal computer connected with a servomotor controller (ez-SERVO, Fastech, Korea) and a custom-made control software program (LabVIEW, National Instruments, Austin, TX, USA). The rotation shaft of the servo motor was coupled to a needle holder (Figure 1D). A rubber grommet was fixed to the needle at a distance of 2 or 3 mm from the tip, as described previously (Kim et al., 2013), to control the depth of acupuncture needle insertion.

In the rat model of cocaine-induced locomotor activity, acupuncture needles (0.10 mm in diameter, needle length of 10 mm and handle length of 10 mm; Dongbang Medical Co., South Korea) were inserted perpendicularly into the HT7 acupoint at a depth of 3 mm, twisted for 20 s with the RANT device, maintained in place for up to 60 s after needle insertion and withdrawn. In the animal model of immobilization-induced hypertension, needles were inserted perpendicularly into the PC6 acupoint at a depth of 3 mm, twisted for 10 min with the RANT device and withdrawn. In the rat model for mustard oil-induced visceral pain, acupuncture needles were inserted 2–3 mm deep into BL62-64 and manually twisted for 30 s at 10 min intervals, which was repeated 4 times for a total of 30 min. Type I collagenase or bupivacaine was injected into acupoints 30 min before acupuncture treatments.

### Cocaine-Induced Locomotor Activity

Locomotor activity was measured through a video tracking system (EthoVision, Noldus Information Technology BV, Netherlands). Briefly, in a dimly lit room, each animal was placed in a square open field box (40 × 40 × 45 cm) made of black acrylic. Video tracking software (EthoVision 3.1) measured the distance traveled (cm). On the testing day, animals were habituated for at least 60 min. After baseline activity was recorded for 30 min, the animal received an intraperitoneal injection of cocaine (15 mg/kg) and acupuncture treatment and was monitored for up to 60 min after injection. Data are reported as the total distance traveled (cm) in 1 h or the distance traveled (cm) during each 10 min.

### Immobilization-Induced Hypertension and Measurement of Blood Pressure

Hypertension was induced by restraining in animals a cone-shaped plastic bag, as described previously (Kim et al., 2017). Systolic blood pressure was measured noninvasively with a tail cuff blood pressure monitor (Model 47, IITC). Briefly, the restrained rats were placed in a chamber kept at 27°C, and an occluding cuff and a pneumatic pulse transducer were positioned on the base of the rat tail. A programmed electrosphygmomanometer (Narco Bio-Systems Inc., USA) was inflated and deflated automatically, and the tail cuff signals from the transducer were automatically collected every 10 min using an IITC apparatus (Model 47, IITC Inc., USA). The mean of the two readings was taken at each blood pressure measurement.

### Mustard Oil-Induced Visceral Pain and Measurement of Electromyographic Response to Colorectal Distension

A pair of silver wire electrodes (0.20 mm in diameter, Nilaco, Japan) were inserted into the external oblique abdominis muscle, buried for 10 mm of their length and separated by 10 mm for electromyography (EMG) recording. Colorectal distension (CRD) was applied using an inflatable balloon (3 cm in length, 1 cm in diameter) attached to an intravenous line via a T connector that was connected to a bulb with a valve and a sphygmomanometer. EMG recordings in response to CRD stimulation for 5 s at strengths of 20, 40, 60, and 80 mmHg were carried out. The EMG signal was amplified (×10,000, 300–1,000 Hz; ISO-80, World Precision Instruments, USA), digitized and analyzed with a data acquisition and analysis interface (Micro1401, CED, UK). After measurement of baseline, for induction of colonic pain, a mustard oil (30~50 μl; Sigma Aldrich)-soaked Q-tip was inserted into the colon, approximately 5 cm proximal to the anus, kept for 30 min and withdrawn. Acupuncture was then applied, and EMG was recorded up to 30 min after acupuncture treatment.

### Measurement of Needle Rotational Force

The rotational force (torque) acting on the needle in the tissue was measured through a custom-made needle force measurement system. The torque sensor was simply constructed by fixing a strain gauge (KFH-C1 Series, OMEGA Engineering, USA) to a plunger of a 50 ml syringe connected to a needle holder (Figure 4A) and wired to a Wheatstone bridge. The signals generated from the torque sensor during needle twisting were digitalized using a data acquisition system (DAQ-NI USB-6200, National Instruments, USA) and analyzed with a customized LabVIEW program (National Instruments; Figure 4B).

### Hematoxylin-Eosin (H&E) Staining and Immunohistochemical Staining of PGP9.5 Expression in the Skin of Type I Collagenase-Treated Rats

At the end of the experiments, the rats were sacrificed under sodium pentobarbital (90 mg/kg, IP) anesthesia. The skin (10 × 10 × 10 mm) over the PC6 acupoints injected with either type I collagenase or bupivacaine was removed and fixed in 4% buffered paraformaldehyde (PFA) for 2 h. The samples were processed, embedded in paraffin wax, and cut into 5-μm-thick serial sections. The skin samples were subjected to H&E staining or immunohistochemistry for PGP9.5. For immunohistochemistry of PGP9.5, the sections were incubated with anti-PGP9.5 mouse monoclonal antibodies (1:1000; Abcam, Cambridge, UK), followed by incubation with a biotinylated donkey anti-mouse Alexa Fluor 488 (1:200; Invitrogen, USA). Images were taken from 4–5 sections from each animal under an epifluorescence microscope (AX70, Olympus, USA) and analyzed with ImageJ software (NIH, USA). A mixture of type I collagenase and toluidine blue (20 μl/locus) was injected into the PC6, HT7, and BL62 acupoints to a depth of 3 mm in a normal rat to further evaluate the extent of the diffusion of type I collagenase after the injection. Thirty minutes after the injection, the tissues were removed, post-fixed, protected with sucrose, and cryosectioned at a thickness of 15 μm. The blue-stained areas were examined under a microscope.

### Data Analysis

All data are presented as the mean ± SEM (standard error of the mean) and analyzed by one- or two-way repeated measurement analysis of variance (ANOVA) followed by post hoc testing using Fisher's least significant difference (LSD) method or paired or unpaired *t*-test, where appropriate. Statistical significance was considered at *P* < 0.05.

## Results

### Construction of a Robotic Acupuncture Needle Twister (RANT) That Mimicked Human Needle Twisting Manipulation

To evaluate the rotational duration (speed) and angle generated during needle twisting, 3 well-trained acupuncturists were asked to manually twist the acupuncture needle (Figure 1A), video-recorded and analyzed using media player software. Across 58 instances, the needles were rotated for an average of 0.972 ± 0.034 s (*n* = 30) in a clockwise direction (CW), left stationary for an average of 0.035 ± 0.001 s (*n* = 52) and then rotated for an average of 0.936 ± 0.028 s (*n* = 28) in an anticlockwise direction (ACW) (Figure 1B). The angles of rotation were 356° ± 4.37° CW and 322.32° ± 6.19° ACW (Figure 1C). However, there were no significant differences in rotation duration or angles between CW and ACW.

To control the rotation speed and angle objectively, a robotic twister was constructed with a servo motor and interfaced with a LabVIEW program written by our laboratory (Figure 1D). When the above parameters for the device were set, the robotic twister replicated the motion of human acupuncture needle manipulation (Supplementary Movie 1).

### Twisting Acupuncture Suppressed Cocaine-Induced Locomotor Activity

**Figure 2:**
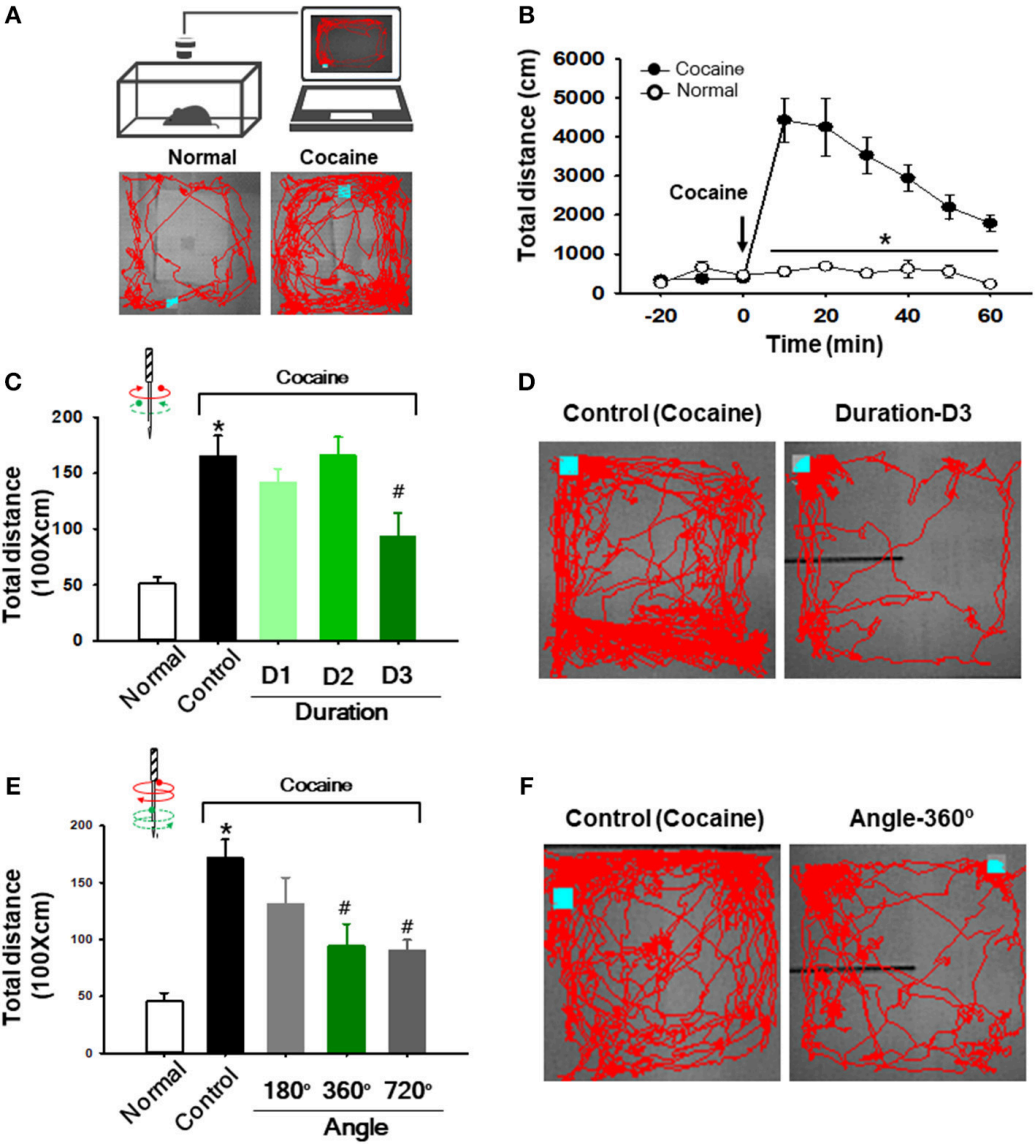
Effect of twisting acupuncture by RANT on cocaine-enhanced locomotor activity. **(A)** Schematic for measurement of locomotor activity (upper) and representative tracings in normal or cocaine-treated rats (lower). **(B)** Enhancement of locomotor activity following intraperitoneal injection of cocaine (*n* = 7) compared to normal rats (*n* = 5). ^*^p < 0.05 vs. Normal. **(C,D)** Effect of acupuncture at various rotation durations on cocaine-induced locomotion. Needle twisting at Duration-D3 significantly decreased cocaine-induced locomotion compared to the control group [^*^p < 0.05 vs. Normal; ^#^*p* < 0.05 vs. Control (cocaine only without acupuncture); Normal, *n* = 5; Control, *n* = 7; Duration-D1, *n* = 8; Duration-D2, *n* = 7; Duration-D3, *n* = 6). **(E,F)** Effect of various needle rotation angles by RANT on cocaine-induced locomotion. Inhibition of cocaine-induced locomotion was found in 360° or 720° rotation angle groups compared to control (cocaine only). (^*^p < 0.05 vs. Normal; ^#^*p* < 0.05 vs. Control; *n* = 6 per group).

To test whether the effect of acupuncture effect is dependent on the needle rotation duration (and speed), we evaluated the effects of needle rotation at various durations on cocaine-enhanced locomotor activity. After baseline recording of locomotor activity for 30 min, rats were given an intraperitoneal (IP) injection of cocaine (15 mg/kg). One minute after injection, acupuncture needles were inserted into the bilateral HT7 and twisted for 20 s at different rotation durations (Table 1) using the robotic twister. Control rats received the cocaine injection without acupuncture treatment. An acute cocaine injection rapidly increased locomotor activity, which lasted up to approximately 60 min after injection, with a peak at 10 min (cocaine group in Figures 2A,B). Acupuncture applied with the Duration-D3 condition (fastest rotation speed) significantly decreased cocaine-induced locomotion compared to the control group [one-way ANOVA, *F*_(3, 21)_ = 3.706; ^#^*p* < 0.05 vs. Control; Figures 2C,D]. However, needle twisting at other durations (Duration-D1 and D2) or needle placement without twisting (Supplementary Figure 1) failed to inhibit the cocaine-induced increase in locomotor activity, suggesting that the inhibitory effects were not stimulus-duration dependent. To determine whether the inhibitory effects of twisting acupuncture are dependent on the needle rotation angle, rats (*n* = 29) were randomly divided into the following groups: control and 180°-, 360°-, and 720°-rotation angle groups (Table 2). Although acupuncture stimulation tended to decrease the locomotor response to cocaine in an angle-dependent manner, significant inhibitory effects of acupuncture on locomotor activity were found in the 360°- and 720°-rotation angle groups compared to the control [one-way ANOVA, *F*_(3, 21)_ = 4.998; ^#^*p* < 0.05 vs. Control; Figures 2E,F]. Because a twisting condition of Duration-D3 produced consistent, reproducible acupuncture effects, the parameter was used in subsequent experiments.

**Table 1:** Parameters for various rotational durations by robotic twister.

**Group**	**Clockwise (CW; s)**	**Pause (s)**	**Anti-clockwise (ACW; s)**	**Rotation (°)**
Duration D-1	3.80	0.035	3.80	360
Duration D-2	1.90	0.035	1.90	360
Duration D-3	0.95	0.035	0.95	360

**Table 2:** Parameters for various rotation angles produced by the robotic twister.

**Group**	**Clockwise (CW; s)**	**Pause (s)**	**Anti-clockwise (ACW; s)**	**Rotation (°)**
180°	0.475^*^	0.035	0.475^*^	180
360°	0.95^*^	0.035	0.95^*^	360
720°	1.90^*^	0.035	1.90^*^	720

* *Note that rotational speeds (time/turn) were the same in all groups*.

### Acupuncture Effects Were Prevented by Blockade of the Nerve but Not Disruption of Connective Tissue

**Figure 3:**
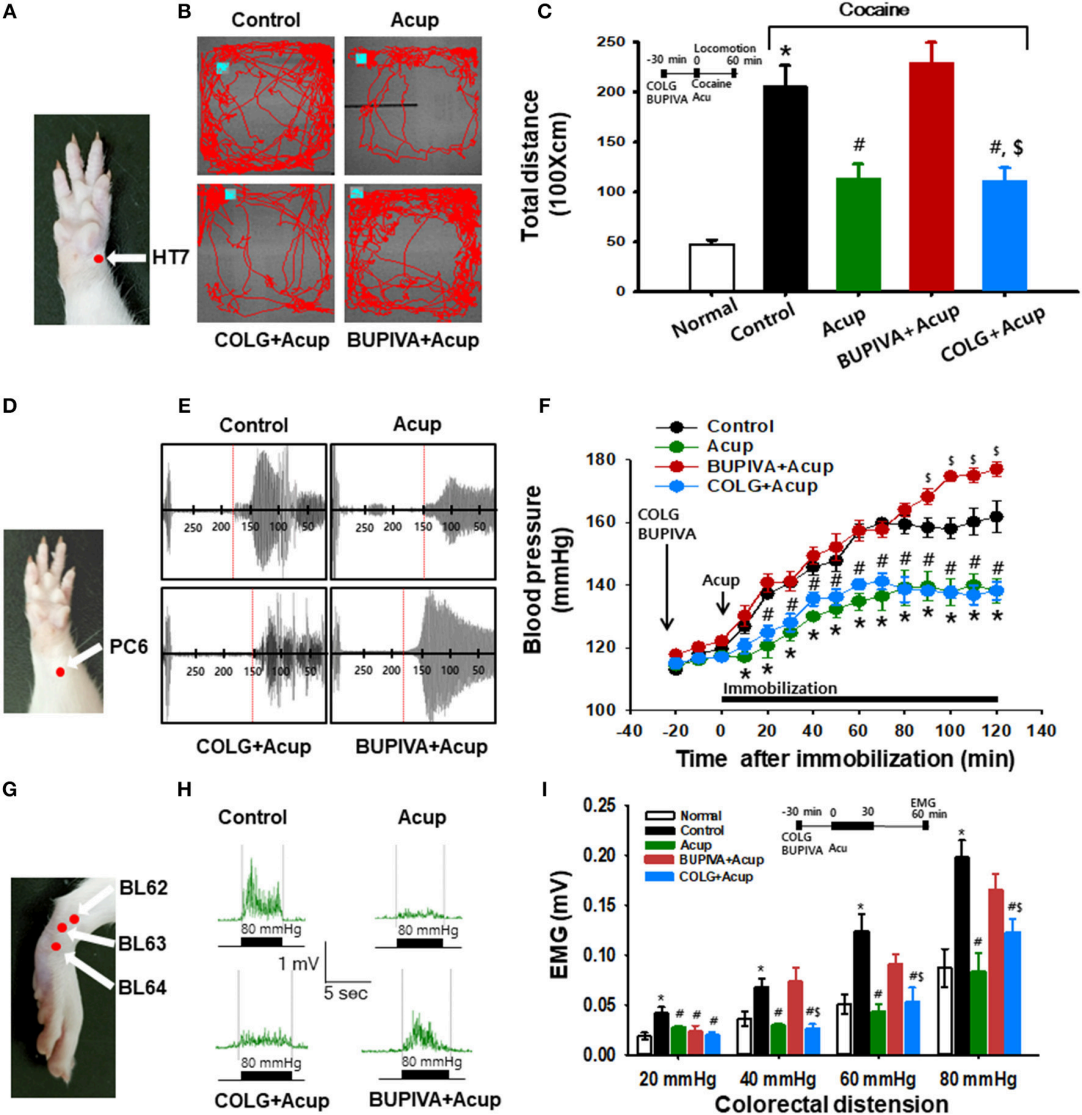
Effect of preinjection of collagenase or bupivacaine into acupoints in various animal models. **(A–C)** Effect of pretreatment of type I collagenase or bupivacaine in HT7 acupoint on cocaine-induced locomotor activity in rats. Inhibition of cocaine-induced locomotion by acupuncture at HT7 (Acup) was prevented by pretreatment of bupivacaine (BUPIVA) into HT7 acupoint **(A)**, but not type I collagenase (COLG; **B,C**). ^*^p < 0.05 vs. Normal; ^#^*p* < 0.05 vs. Control; ^$^*p* < 0.05 vs. BUPIVA+Acup; *n* = 5 per group. Representative tracings **(B)**. **(D–F)** Effect of pretreatment of type I collagenase or bupivacaine in the PC6 acupoint on immobilization-induced hypertension in rats. Bupivacaine, but not collagenase, inhibited the acupuncture effects of PC6 **(D)** on elevated systolic blood pressure in rats **(E,F)** Representative pulse signals measured from tail **(E)** ^*^p < 0.05 vs. Control (immobilization only without acupuncture); ^#^*p* < 0.05 vs. BUPIVA+Acup; *n* = 5 per group. **(G–I)** Effect of pretreatment of type I collagenase or bupivacaine in acupoints on mustard oil-induced visceral pain in rats. Electromyogram (EMG) in each group was recorded during colorectal distension of 20, 40, 60, and 80 mmHg for approximately 5 s. Normal, normal rats; Control, mustard only without acupuncture; Acup, acupuncture in mustard-treated rats; COLG+Acup, pretreatment of collagenase prior to acupuncture in mustard-treated rats; BUPIVA+Acup, pretreatment of bupivacaine prior to acupuncture in mustard-treated rats. *n* = 7 per group. Acupoints used. **(G)** Representative EMG signals recorded during colorectal distension of 80 mmHg **(H)**. Bupivacaine, but not collagenase, inhibited the acupuncture effects on colorectal distension-induced visceral motor responses in rats **(I)**. ^*^p < 0.05 vs. Normal; ^#^*p* < 0.05 vs. Control; ^$^*p* < 0.05 vs. BUPIVA+Acup; *n* = 7 per group. Acup, acupuncture; COLG, collagenase; BUPIVA, bupivacaine.

To investigate whether acupuncture effects are mediated via nerve or connective tissue, we injected either bupivacaine (a local anesthetic) or type I collagenase (a proteolytic enzyme) into acupoints 30 min before acupuncture treatment. The experiment was first performed in the rat model of cocaine locomotion. While acupuncture at HT7 attenuated cocaine-induced enhancement of locomotor activity, such effects were inhibited by pretreatment of bupivacaine, but not collagenase, indicating mediation of the afferent nerve [one-way ANOVA, *F*_(3, 21)_ = 4.998; ^*^p < 0.05 vs. Normal; ^#^*p* < 0.05 vs. Control; ^$^*p* < 0.05 vs. BUPIVA+Acup; Figures 3A–C]. To further confirm this, we repeated the experiments in the rat models of immobilization-induced hypertension (IMH) or mustard oil-induced visceral pain. For the IMH model, when a rat was placed in an immobilization bag, systolic blood pressure (SBP) gradually increased for the next several hours (Control; Figures 3D–F), consistent with our previous study (Kim et al., 2017). Twisting acupuncture at PC6 with the robotic device prevented the development of hypertension compared to the control (^*^p < 0.05 vs. Control; Figure 3F). When either bupivacaine or collagenase was injected into PC6 30 min before acupuncture treatment, the acupuncture effects were prevented in bupivacaine-treated rats but not in collagenase-treated rats (Figures 3E,F). Unexpectedly, significant differences were observed at the time points of 100–120 min between the control and BUPIVA+Acup groups (^$^*p* < 0.05 compared to the control group; Figure 3F). Compared to our previous study (Kim et al., 2017), 2 of 5 control rats developed borderline hypertension (140–160 mmHg) and did not reach an SBP of 160–180 mmHg at the end of the 2 h immobilization, which would yield a significant difference at the later time points between the control and Bupiva+Acup groups. In the rat model of mustard oil-induced visceral pain, acupuncture needles were inserted into BL62-64 and manually rotated, and visceral sensitivity was assessed in recording the electromyographic (EMG) responses to graded colorectal distensions (CRD) of 20, 40, 60, and 80 mmHg in conscious rats. A significant increase in the EMG discharges in response to CRDs was observed in mustard oil-treated rats (Control) compared to normal rats (Normal). The elevated EMG responses were attenuated in the rats given acupuncture treatment [one-way ANOVA, 20 mmHg; *F*_(4, 30)_ = 4.755; ^#^*p* = 0.025, 40 mmHg; *F*_(4, 30)_ = 7.273; ^#^*p* = 0.003, 60 mmHg; *F*_(4, 30)_ = 8.047; ^#^*p* < 0.001, 80 mmHg; *F*_(4, 30)_ = 8.845; ^#^*p* < 0.001 vs. Control], which was reversed by pretreatment of bupivacaine [one-way ANOVA, 20 mmHg; *F*_(4, 30)_ = 4.755; *p* = 0.514, 40 mmHg; *F*_(4, 30)_ = 7.273; *p* < 0.001, 60 mmHg; *F*_(4, 30)_ = 8.047; *p* = 0.009, 80 mm Hg; *F*_(4, 30)_ = 8.845; *p* = 0.002 vs. Acup]. However, the inhibitory effects of acupuncture on visceral pain were not altered by treatment with collagenase prior to acupuncture treatment (Figures 3G–I). This observation indicates the mediating role of afferent nerves in the effects of acupuncture.

### Type I Collagenase Disrupted Connective Tissue in Acupoints Without Morphological Changes in the Afferent Nerve

**Figure 4:**
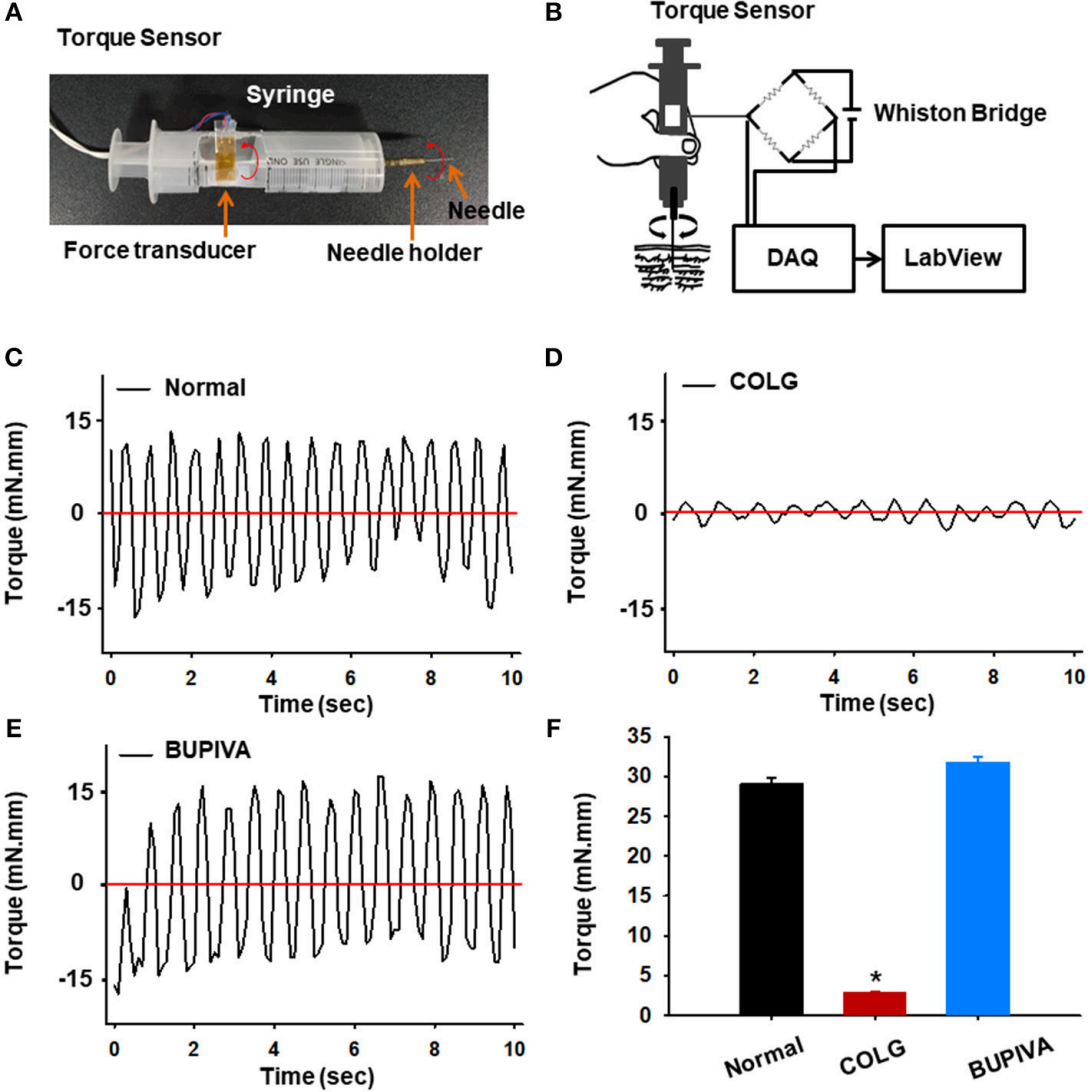
Effect of preinjection of collagenase or bupivacaine into acupoints on rotational force (torque). **(A,B)** A simply constructed torque sensor **(A)** and its electrical circuit **(B)**. **(C–F)** Rotational force recorded during needle twisting in collagenase or bupivacaine-treated acupoints. A significant drop of rotational force was found in collagenase-treated acupoints **(D)** compared to normal **(C)** or bupivacaine-treated **(E)** rats. ^*^p < 0.05 vs. Normal; *n* = 5 per group. COLG, collagenase; BUPIVA, bupivacaine.

To assess the functional alteration of connective tissue in the acupoints injected with collagenase or bupivacaine, we constructed a torque sensor (Figures 4A,B) and measured rotational force (torque) acting on the needle in the tissue. The acupuncture needle inserted into the HT7 acupoint over the wrist was connected to the needle holder of the torque device and manually twisted. The bidirectional needle twisting generated sawtooth waves, with amplitudes in the range of −15 to 15 mN.mm in the untreated normal rats (Normal, Figure 4C). While needle rotational force was not changed in bupivacaine-injected acupoints compared to those of normal rats (BUPIVA; Figures 4E,F), a significant drop in rotational force was found in collagenase-treated acupoints (COLG; ^*^p < 0.05 vs. Normal; Figures 4D,F). This observation indicates that type I collagenase eliminated the pulling of collagen fibers created during needle manipulation.

**Figure 5:**
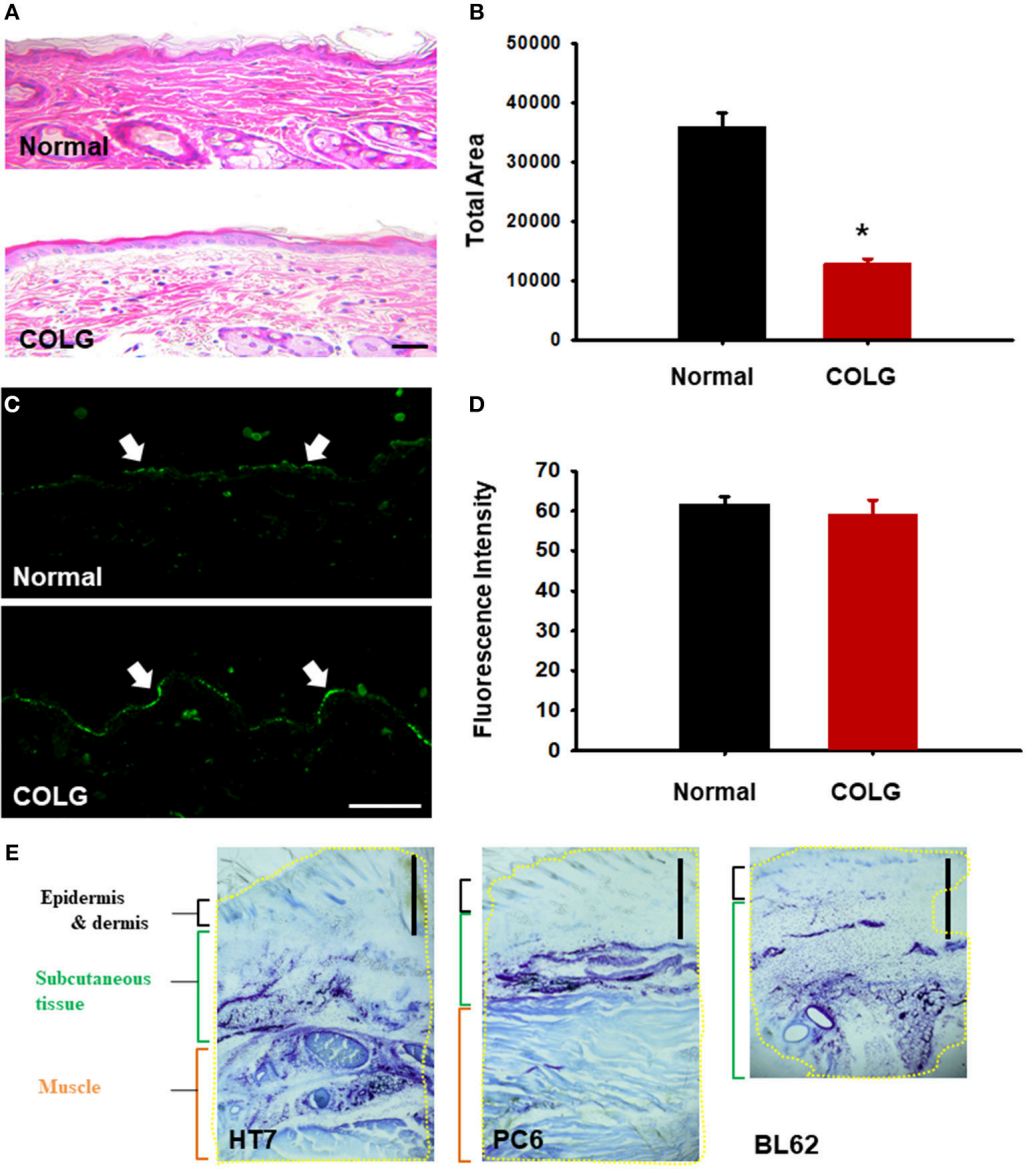
Histological or immunohistochemical examination of collagenase-injected acupoints. **(A,B)** H&E staining of collagenase- or saline-treated acupoints. Quantification of pink-stained area in normal or collagenase-treated skins **(B)**. ^*^p < 0.05 vs. Normal; *n* = 5 per group. **(C,D)** Immunohistochemistry for PGP9.5, a neuronal marker, in normal or collagenase-treated acupoints **(C)**. Mean fluorescent intensity in normal or collagenase-treated skins **(D)**. *n* = 5 per group. COLG, collagenase. Bar = 50 μm. **(E)** Diffusion of a mixed solution of toluidine blue dye and type I collagenase injected into acupoints. Yellow line = blue area stained with toluidine blue. Bar = 1 mm.

To identify whether collagenase causes morphological damage of connective tissue or peripheral nerve in the acupoints, the skin over acupoints was evaluated through histological or immunohistochemical methods. In H&E staining, the skin treated with collagenase displayed faint staining, with sparse thin collagen bundles and a significantly decreased density of collagen fibers compared to that of normal, untreated rats (Figures 5A,B; ^*^p < 0.05). On the other hand, in a quantitative immunohistochemical analysis for PGP 9.5, a marker for nerve fibers (Beiswenger et al., 2008), the morphological changes of the peripheral nerve were not observed in collagenase-treated skin compared to the normal group (Figures 5C,D). In addition, the histological examination revealed that the type I collagenase that had been injected into the acupoints diffused into the skin, including the epidermis, dermis, and subcutaneous tissues (Figure 5E). This suggests that injection of type I collagenase into acupoints disrupted the connectivity of collagen fibers but did not induce morphological damage of peripheral nerves in skin.

## Discussion

Using our robotic device that mimicked needle twisting by a human acupuncturist, we demonstrated that twisting acupuncture effectively suppressed cocaine-induced locomotor activity, immobilization-induced hypertension and mustard oil-induced visceral pain, which was completely prevented by blocking afferent nerves with bupivacaine but not by degrading connective tissue with type I collagenase. Furthermore, collagenase destroyed collagen in the skin without damaging the peripheral nerve. Our findings suggest critical mediation of the nerve in producing acupuncture effects. Additionally, modulation of connective tissue does not seem to be sufficiently reliable to serve as the principal explanation for acupuncture effects, at least in the animal models tested.

Acupuncture needles are manually manipulated to obtain therapeutic effects after needle insertion into acupoints (Pomeranz and Berman, 2003). Various manipulation techniques, such as twisting (also known as bidirectional twisting-rotation), vibrating and lifting and thrusting, have been used in clinics. Among them, manual twisting has been popular because it is a simple and effective technique to provoke the needling sensation called *deqi*, which acupuncturists believe to be essential for obtaining effective acupuncture stimulation (Pomeranz and Berman, 2003; Stux and Pomeranz, 2012). (Kim G. H. et al., 2010) reported that manual twisting is more effective than the lifting-thrusting technique in producing analgesic effects on formalin-induced pain in rats. We and others have shown that manual twisting of needles applied to HT7 acupoints suppresses addictive behaviors induced by abused drugs, including cocaine, methamphetamine, and ethanol (Yang et al., 2010; Jin et al., 2018; Kim et al., 2018). Moreover, acupuncture at HT7 attenuates drug-induced dopamine release and metabolic neuronal activity in the nucleus accumbens (Yoon et al., 2004; Jin et al., 2018) through group II metabotropic glutamate receptors (Kim et al., 2018) and modulation of GABA neuron activity in the ventral tegmental area (Yang et al., 2010). While these studies provide evidence that twisting acupuncture needle manipulation produces therapeutic effects, manual acupuncture stimulation could alter its effect by affecting many vagaries, including rotation durations and angles. Unfortunately, few studies have quantified the twisting technique performed by practitioners. Therefore, the present study video-analyzed the motion of needle twisting performed by acupuncturists and found that the duration of needle rotation ranged from 0.75 to 1.21 s (mean value of 0.975 ± 0.073 s) and rotation angles ranged from 325° to 350° (mean value of 340° ± 4.27°). However, considerable variations in both durations and angles were observed within each acupuncturist or across acupuncturists (data not shown). The variations may lead to low reproducibility and high individual variations among practitioners (Napadow et al., 2005; Kim et al., 2013). As our robotic twister has an advantage in controlling rotation duration and angle, it would help solve the control issues of manual needle manipulation.

Researchers have suggested that needle twisting in different stimulation parameters [including durations (or frequency, Hz) and angles, etc.] could produce different physiological responses and therapeutic effects (Kim G. H. et al., 2010; Chang et al., 2017). Hong et al. (2015) showed that the responses of spinal dorsal horn neurons following gastric distension are enhanced by manual acupuncture at 0.5 and 1 Hz, while the dorsal horn neurons are inhibited by manual acupuncture at 2 and 3 Hz. In the present study, the effects of acupuncture were not dependent on the rotation duration or angle when various durations or angles of needle twisting were tested in three different animal models. Similarly, a previous study attempted to determine the analgesic effects of acupuncture in 4 different stimulation conditions (angle and frequency of rotation: 90° + 1 Hz, 90° + 1/4 Hz, 360° + 1 Hz, and 360° + 1/4 Hz) by using a computer-controlled rotational device in rats. This study showed that only the 90° + 1/4 Hz condition generates a statistically significant effect compared to plain acupuncture (Kim et al., 2006). These findings indicate that there does not appear to be a correlation between rotation durations or angles and the degree of response alteration in the twisting needle manipulation. Rather, acupuncture effects may require specific stimulation parameters, in accordance with what has been found in animal models. Additionally, in accordance with a previous study (Kim et al., 2006), our data showed that simple needle placement did not generate acupuncture effects on cocaine-induced locomotion in rats. It supports the observations that needle manipulations such as rotation and electrical stimulation are needed to achieve the desired effects by activating sensory nerve fibers and cells, in contrast to needle placement (sham acupuncture) (Kim et al., 2006; Guo et al., 2018).

In the present study, injection of a long-lasting local anesthetic bupivacaine into acupoints effectively blocked acupuncture effects in the rat models of cocaine-induced locomotion, hypertension, and mustard oil-induced visceral pain, suggesting that acupuncture signals are conveyed via afferent nerve fibers. These results agree with reports that application of a local anesthetic, 1% lidocaine, to acupoints prior to acupuncture treatment inhibits antiemetic action by acupuncture at PC6 in patients undergoing gynecological surgery (Dundee and Ghaly, 1991), and that injection of a local anesthetic procaine into ST36 blocks acupuncture analgesia in animal models (Ulett et al., 1998). Additionally, the role of afferent nerve fibers in acupuncture-initiated impulses was supported by several observations (given below). Acupuncture analgesia is reported to be ablated after sectioning nerve innervating acupoints (Noguchi and Hayashi, 1996; Kim H. Y. et al., 2010) and is reproduced by direct stimulation of exposed nerves (Li et al., 1998; Kim H. Y. et al., 2010). Manual and electroacupuncture at PC6-7 acupoints activate Aδ and C fibers to evoke a cardiovascular effect (Zhou et al., 2005), which is eliminated by neonatal capsaicin to block C-fiber afferents (Tjen-a-Looi et al., 2005). The acupoint stimulation activates opioid receptors in the rostral ventrolateral medulla, a central site regulating blood pressure (Sved et al., 2003), and suppresses stimulation-induced hypertension (Tjen-a-Looi et al., 2003; Kim et al., 2017). As shown in our previous study, mechanical stimulation of HT7 acupoints activates peripheral sensory afferents, such as Pacinian and Meissner's corpuscles, which are conveyed via large A-fibers of the ulnar nerve and spinal dorsal column somatosensory pathway. These signals enhance γ-aminobutyric acid (GABA) neuronal activity in the ventral tegmental area (VTA) and suppress extracellular dopamine release in the nucleus accumbens (Jin et al., 2018), resulting in the attenuation of cocaine-induced locomotor activity or cocaine-seeking behaviors (Kim et al., 2013; Chang et al., 2017; Jin et al., 2018). Indeed, the above evidence indicates that acupuncture stimulates afferent nerve fibers which, in turn, send messages to the brain.

An emerging theory proposes a mediating role of connective tissue in the effects of acupuncture, wherein acupuncture transmits signals through the layers of connective tissue surrounding muscle groups, organs, and blood vessels. This theory posits that acupuncture needle manipulation creates winding of connective tissue around the needle, known as “needle grasp,” and in turn activates signal pathways by deformation of connective tissue, resulting in therapeutic effects (Langevin and Yandow, 2002; Langevin et al., 2007). Previous studies have also found that needle twisting produces an active cytoskeletal response in a manner dependent on the number of rotations and the angle of the needle (Langevin et al., 2007) This procedure also induces degranulation of the mast cells in the tissues of acupoints through an interaction with the collagen fibers (Zhang et al., 2008; Wu et al., 2015). Although many studies, mostly led by the Langevin research group, have proposed biomechanical actions of connective tissue during acupuncture manipulation (Langevin and Yandow, 2002; Langevin et al., 2007; Langevin, 2014), to date, it has not been determined fully whether the connective tissue is associated with therapeutic effects of acupuncture. In the present study, a peak-to-peak torque value of about 30 mN.mm was generated by twisting a needle inserted into HT7, which was decreased to about 3 mN.mm after injection of type I collagenase, similar to those reported in a previous study (Yu et al., 2009). Thus, an injection of type I collagenase into acupoints disrupted the phenomenon of “needle grasp” by causing connective tissue breakdown. Type I collagenase did not affect the expression of the neuronal marker PGP9.5 in skin, indicating that the afferent nerve in the collagenase-treated skin was intact. Importantly, disruption of connective tissue by type I collagenase failed to block the acupuncture effects generated by needle twisting in the rat models tested. On the other hand, bupivacaine, a long-acting local anesthetic (Rosenberg and Heinonen, 1983), injected into acupoints effectively blocked the acupuncture effects without affecting needle grasp of connective tissue. Taken together, our data suggest that acupuncture may not recruit connective tissue to produce therapeutic effects. In contrast to our results, Yu et al. reported that the destruction of collagen fibers with type I collagenase at ST36 acupoints inhibited the effects of acupuncture on inflammatory pain in rats (Yu et al., 2009). The reason for this discrepancy is not completely understood at present, but a possible explanation is that the acupuncture points chosen in this experiment were located on distal limbs (HT7, PC6, and BL62-64), which contain relatively less subcutaneous tissue, while the experiments in the paper by Yu et al. used ST36, which is a more proximal acupoint enriched in loose connective tissue. Considering that the interaction of the needle with collagen fibers mainly occurs within the loose subcutaneous tissue, but not within the dermis (Langevin and Yandow, 2002), the interaction between the needle and loose connective tissue in proximal acupoints such as ST36 may be greater than the distal acupoints used in the current study. However, our ability to draw conclusions about the importance of afferent nerve fibers in acupuncture-mediated responses, rather than connective tissue, was hampered by the major limitation of the present study, in that 5 points of approximately 360 acupoints were investigated using three animal models. More extensive studies using various animal models and acupoints will be required.

In conclusion, twisting acupuncture produced therapeutic effects in rat cocaine-induced locomotion, hypertension and mustard oil-induced visceral pain models, and these effects were abolished by a blockade of the afferent nerve in the vicinity of acupoints but not by disruption of collagen fibers. Accordingly, it seems reasonable to propose that the effects of acupuncture require mediation by nerve tissue but not connective tissue. This study helps to understand the mechanisms involved in the initiation of acupunctur signals.

## Author Contributions

HYK and CY designed the experiment. SC, YR, SB, JB, YF, OK, MB, D-HK, SL, HKK, and BL conducted the experiments. HYK was responsible for the overall direction of the project and for edits to the manuscript.

### Conflict of Interest Statement

The authors declare that the research was conducted in the absence of any commercial or financial relationships that could be construed as a potential conflict of interest.

## Supplementary Material

Supplementary Movie 1 Needle twisting by a robotic acupuncture needle twister or an acupuncturist.
