# Supplementary material for: Peripheral Sensory Nerve Tissue but Not Connective Tissue Is Involved in the Action of Acupuncture
Source: Front Neurosci. 2019 Feb 20;13:110. doi: 10.3389/fnins.2019.00110 (PMC6401607; doi:10.3389/fnins.2019.00110)
Supplement: Supplementary file 2 [file Image_1.pdf]

Supplementary Material

1. Supplementary Figures

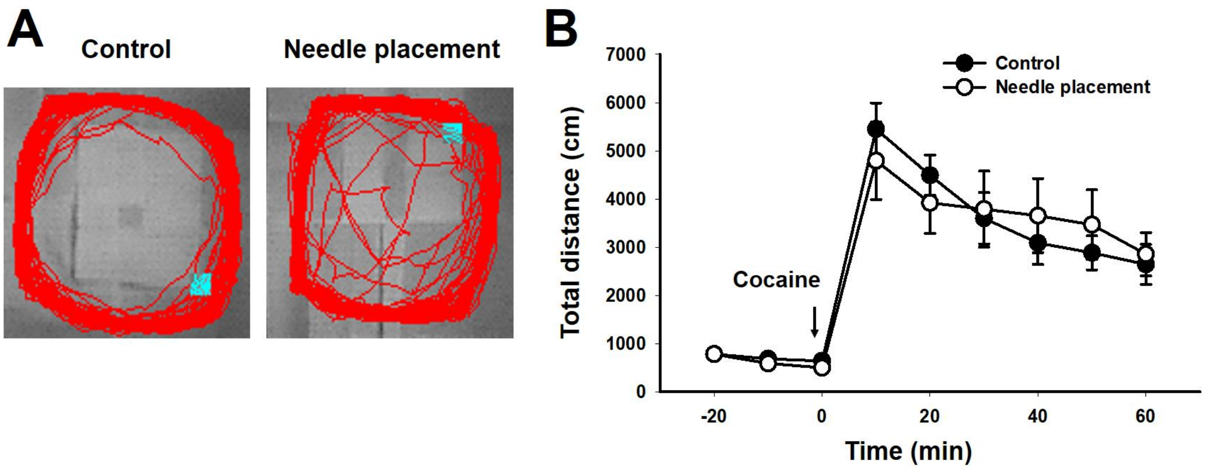

Supplementary Figure 1. Effect of needle placement on cocaine-enhanced locomotor activity.

A. Representative locomotor activity traces following cocaine injection in control and needle placement groups. **B**. The rats given needle insertion into HT7 without needle twisting (Needle placement; n=5) did not show any inhibitory effects on cocaine-induced locomotor activity, compared to control (n=5, cocaine only)
